# Maternal Perinatal Depression and Risk of Neurodevelopmental Disorders in Offspring: Preliminary Results from the SOS MOOD Project

**DOI:** 10.3390/children8121150

**Published:** 2021-12-07

**Authors:** Martina Siracusano, Assia Riccioni, Leonardo Emberti Gialloreti, Elisa Carloni, Antonia Baratta, Marialaura Ferrara, Lucrezia Arturi, Giulia Lisi, Ilaria Adulti, Rodolfo Rossi, Alessia Lucaselli, Alessandro Rossi, Cinzia Niolu, Luigi Mazzone

**Affiliations:** 1 Department of Biomedicine and Prevention, University of Rome Tor Vergata, Via Montpellier 1, 00133 Rome, Italy; leonardo.emberti.gialloreti@uniroma2.it; 2 Child Neurology and Psychiatry Unit, Department of Neurosciences, Policlinico Tor Vergata Foundation Hospital, Viale Oxford 81, 00133 Rome, Italy; assiariccioni@gmail.com (A.R.); elisacarloni2901@gmail.com (E.C.); antoniabaratta@gmail.com (A.B.); marialaura.ferrara93@gmail.com (M.F.); lucrezia.arturi@gmail.com (L.A.); luigi.mazzone@uniroma2.it (L.M.); 3 Systems Medicine Department, University of Rome Tor Vergata, Montpellier Street 1, 00133 Rome, Italy; giulia.lisi@aslroma1.it (G.L.); rudy86.rossi@gmail.com (R.R.); niolu@med.uniroma2.it (C.N.); 4 Mental Health Department, Azienda Sanitaria Locale Roma 1, 00133 Rome, Italy; 5 Psychiatry and Clinical Psychology Unit, Department of Neurosciences, Policlinico Tor Vergata Foundation Hospital, Viale Oxford 81, 00133 Rome, Italy; ilaria.adulti@gmail.com; 6 Section of Psychiatry, Department of Biotechnological and Applied Clinical Sciences, University of L’Aquila, 67100 L’Aquila, Italy; alessialucaselli@gmail.com (A.L.); alessandro.rossi@univaq.it (A.R.)

**Keywords:** perinatal, depression, offspring, neurodevelopment, autism spectrum disorder, antidepressant, pregnancy, pharmacotherapy, SSRI, impact

## Abstract

The latest research is attempting to define whether there may be an association between maternal Perinatal Depression (PD), the use of psychotropic medications during pregnancy, and a higher risk of neurodevelopmental disorders in children, including Autism Spectrum Disorder (ASD). A better understanding of the relation between PD and ASD is a key element to develop early interventions. This study has been developed in the context of the SOS MOOD project. Its aim is to evaluate the possible impact of maternal PD on the child’s cognitive and behavioral phenotype with a focus on ASD. Women included in the project were screened during pregnancy (1st, 2nd trimester) for PD—categorized as affected or not—and if necessary were prescribed pharmacological therapy; offspring of both groups of women underwent at a mean age of 43 months a standardized neuropsychiatric evaluation of developmental and cognitive skills, behavioral problems, autism symptoms and parental stress. Preliminary results on 59 women and 59 children do not suggest significant long-term effects of maternal PD on offspring’s development and behavior. Nonetheless further studies on wider samples are necessary in order to confirm such results and disentangle the role of possible confounding factors associated to the maternal illness.

## 1. Introduction

Perinatal mental health has become a significant pole of interest in recent years [1], not only for the effects that it may determine on women but also for the impact it may have on offspring [2]. The perinatal period represents in fact a crucial phase for the neurodevelopment of the fetus; and the intra-uterine environment plays a main role in the etiology of Neurodevelopmental Disorders (NDDs) including Autism Spectrum Disorder (ASD) [3], with recent evidence suggesting that the prenatal immune environment may be a particularly promising area for ASD research [4,5].

In particular, maternal inflammation during pregnancy stages induced by several prenatal factors—physical (infections, chronic immune diseases, obesity) and psychological conditions (stress, depression, anxiety)—has been shown to impact on perinatal outcomes and offspring’s development [4,6].

Among perinatal maternal conditions characterized by increased inflammation, there is Perinatal Depression (PD) [7,8], currently defined as a form of depression whose onset occurs during the peripartum period, specifically during pregnancy or within the first 4 weeks of postpartum [9].

Symptoms of perinatal depression are reported in more than 25% of pregnant women and the overt disorder is estimated in the 10–15% [10,11,12], with an increase rate of 51% in comparison to previous generations [13].

Given the increased rates of PD, the necessity of treating and prescribing medications during pregnancy has grown in the last years [14,15,16] with antidepressants representing the first pharmacological choice treatment for major depressive disorders [17,18]. Among these classes of drugs, most women are commonly treated with selective serotonin reuptake inhibitor (SSRIs) even during pregnancy [14,19] although others interrupt the treatment [14]. In fact, even if prenatal prescription and use of SSRI is evaluated as safe, it is debated whether its administration during pregnancy may somehow affect child neurodevelopment with possible negative effects at a bio-behavioral level on offspring [20]. Given the property of SSRIs to cross the placental barrier, they may influence the serotoninergic transmission of the fetus—crucial for brain development—[20,21,22] thus impacting on fetal neurodevelopmental processes and subsequently on behavioral phenotype (i.e., autism symptoms)—as described in animal models [23,24]. On the other hand, untreated perinatal depression could have detrimental effects on the fetus [25].

The main question clinicians and researchers are trying to answer is whether maternal PD and the use of antidepressants during pregnancy have an impact on child development.

What we know until now is that maternal PD seems to have negative effects on both maternal health [26]—weight problems [27], alcohol and illicit drug use [28], breastfeeding problems [29], persistent depression [30], suicidal ideation [31]; and child physical development [26]—preterm births, fetal growth impairments [32] and sleep problems.

Specifically, several studies attempted to examine the possible association between maternal perinatal depression and neurodevelopmental disorders in offspring but with contrasting findings in regard to motor [33,34,35,36] and language development [34,35,36,37,38,39], and more consistent results in terms of behavioral phenotype (i.e., emotional dysregulation, behavioral inhibition, difficult temperament) [40,41,42,43,44,45,46,47].

Specifically concerning the possible association between maternal PD and ASD in offspring, studies focused their attention, although with debatable findings, on prenatal exposure to antidepressants and children’s behavioral outcome [48,49,50,51,52,53,54]. Noteworthy is that most of the research reporting an increased risk of ASD were registry-based studies retrospectively conducted, which did not include a direct evaluation of women in pregnancy and a clinical assessment of children’s development and behavioral outcome through standardized tools.

Among these studies, Croen and colleagues [48], in a population-based control study on 298 children with ASD and 1507 control children, found a 2-fold increased risk of ASD in children exposed to maternal treatment with SSRIs, especially in the first trimester of pregnancy and in the year before delivery. In a more recent work [51] on 254 610 individuals aged 4–17 (5378 with autism), the authors reported that 4.1% of children exposed to maternal use of antidepressants presented a diagnosis of ASD.

To the best of our knowledge two longitudinal studies are actually ongoing [55,56], and even if they include in their protocols a prospective standardized clinical assessment of children, a measure for autism has not been considered.

Most of the literature on the topic reports an increased risk of ASD within offspring exposed to maternal perinatal depression. However, until now no association of causality has been demonstrated between prenatal exposure to antidepressants and ASD and this issue remains an open question [25]. Nevertheless, a better understanding of the relation between perinatal depression and ASD is a key element to develop early interventions intended to progressively modify the resulting developmental trajectories.

These findings collectively highlight the need to define prospectively the clinical characterization of children born from mothers affected by perinatal depression. Therefore, the SOS MOOD project “MOOD of Mothers and Offspring’s Development” has been developed. It is a longitudinal follow-up study of women affected by PD. In such a context we have performed a preliminary study aimed to: (1) evaluate possible long-term effects of maternal perinatal depression on the socio-communicative and behavioral phenotype of the offspring with a specific focus on the possible increase of ASD risk; (2) identify, at a preliminary level, within the subgroup of offspring of mothers with perinatal depression, possible differences in developmental and behavioral profiles, between children exposed (O-PD Treat) or not exposed (O-PD NoTreat) to maternal pharmacological treatment during pregnancy.

Based on the available literature on the topic, an increased risk of neurodevelopmental disorders in offspring exposed to maternal depression during pregnancy might be expected.

## 2. Materials and Methods

The SOS MOOD project is a multicentric screening program for the early detection of maternal perinatal depression and offspring’s cognitive and behavioral development.

It is a mental health safeguard project addressed to women and offspring, developed to support women during pregnancy and post-partum and to early detect child warning signals of a derailed development.

The SOS MOOD project involves: The Italian Psychiatric Units of the University of “Rome Tor Vergata” and “University of L’Aquila”; the Child and Adolescence Psychiatric Unit of the University of “Rome Tor Vergata”. In particular the Psychiatric Units were responsible for, and performed, the screening and clinical assessment for perinatal depression of women during pregnancy; whereas the Child Psychiatry Unit of the University of Rome Tor Vergata performed the neuropsychiatric evaluation of the offspring.

The study protocol has been approved by the Ethical Committees of the Rome Tor Vergata University Hospital (#37/18 March 2018; #145/20 July 2020) and University of L’Aquila, Italy (#123781 November 2020).

All participants provided written informed consent.

### 2.1. Sample and Procedure

#### 2.1.1. Main Procedure

In the context of the SOS MOOD project, the Psychiatric Units of Rome and L’Aquila offer a public service addressed to women perceiving any kind of psychological concern or difficulty during pregnancy.

Pregnant women referring to these Units were specifically screened for perinatal depression (PD) during pregnancy and if necessary were prescribed pharmacological therapy. Furthermore, all women—affected or not by PD—were made aware that a service of child psychiatry was available after delivery, in the case they had any kind of concern about their children’s development.

At a mean distance of 43 months from delivery a multidisciplinary team (child psychiatrists and psychologists) of the Child and Adolescence Psychiatric Unit of the University of Rome Tor Vergata, contacted the women who had been screened by the Psychiatric Units for PD during pregnancy and reminded them about the possibility to benefit from a developmental and behavioral evaluation for their children.

#### 2.1.2. Women

Overall, 488 women referred to the Adult Psychiatric Services during pregnancy in the time range January 2010–January 2020. Out of these 100 mothers were randomly chosen and contacted by the Child and Adolescence Psychiatric Unit, at a mean distance of 43 months from delivery, for the psychiatric evaluation of their offspring. Women with chronic health conditions were excluded, as well as those with implications for immune function, including diabetes, rheumatoid arthritis, multiple sclerosis and/or infectious diseases; twin pregnancies, previa placenta, pre-eclampsia; women pharmacologically treated with drugs others than psychotropic medications prescribed for perinatal depression.

**Figure 1:**
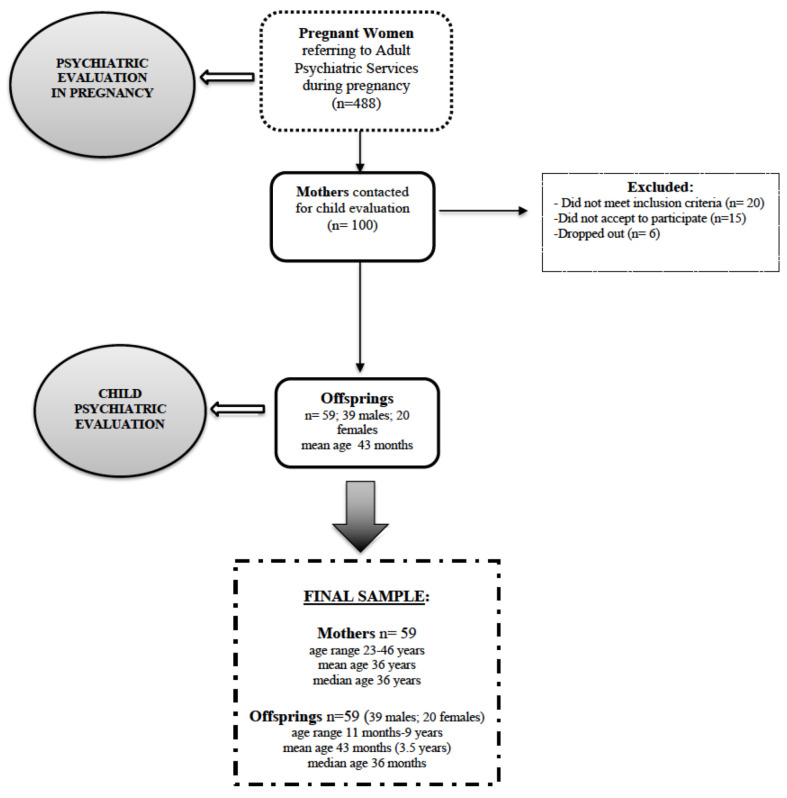
Sample of the study: mothers and their respective offspring.

Out of the 100 selected women, 20 did not meet the inclusion criteria for this research, 15 did not accept to participate, 6 dropped out (did not complete the child evaluation), therefore the final sample of women enrolled was of 59 (mean age 36 years) (Figure 1).

All enrolled women were screened during pregnancy (1st or 2nd trimester) for the presence of perinatal depression. They underwent a clinical diagnostic interview in order to collect demographic and clinical data (i.e., auxological parameters, educational degree, employment, area of residency and habits during pregnancy). The presence of PD was explored during pregnancy by the Psychiatric Units of Rome Tor Vergata and L’Aquila University through clinical evaluation and by the administration of the self-report questionnaire EPDS (Edinburgh Perinatal Depression Scale) [57] (see next paragraph for instrument’s description).

On the basis of the clinical evaluation, the Diagnostic and Statistical Manual of Mental Disorders—Fifth Edition (DSM-5) [9] criteria and the score obtained at EPDS (<12 Not Perinatal Depressed; ≥12 Perinatal Depressed), pregnant women were categorized in:

PD = women with perinatal depression

NPD = women without perinatal depression

#### 2.1.3. Offspring

Children were included in the study at a mean distance of 43 months from delivery, prior to written consent of both parents.

As previously reported, women screened for PD during pregnancy—and turned out to be affected or not by PD—in the context of the SOS MOOD project were aware about the possibility of receiving a psychiatric evaluation of their children. However, mothers were reminded by phone about this opportunity at a mean distance of 43 months from delivery.

Offspring of all the groups of women included in the study underwent a neuropsychiatric evaluation at a mean age of 43 months (Figure 1). A total of 59 children (39 males; 20 females; age range 11 months–9 years, mean age 3.5 years) were finally enrolled in the study. In particular, an assessment of developmental or intellectual quotient, adaptive functioning, autism symptoms, behavioral problems, was performed with the standardized instruments described below. Each measure was administered to participants depending on their chronological age (see the paragraphs below). Finally, at the moment of child neuropsychiatric evaluation, parents underwent an evaluation of parental stress through a self-report questionnaire: The Parental Stress Index Short Form (PSI-SF).

### 2.2. Materials

#### 2.2.1. Women

##### Depressive Symptoms Measures

Edinburgh Postnatal Depression Scale [57].

The presence of depressive symptoms among the studied women was assessed with the Edinburgh Postnatal Depression Scale (EPDS). The EPDS, firstly elaborated in the United Kingdom by Cox, Holden and Sagovsky in 1987 [57], represents one of the most employed screening instruments for depression and anxiety related to pregnancy [26].

It is a self-report 10-item questionnaire which assesses depressed mood, anhedonia, anxiety, and self-harm over the past seven days using a 4 point Likert-scale. The total score ranges from 0 to 30, where higher scores indicate more severe depressive symptoms. The range of cut-off scores selected for detecting depressive symptoms is extremely variable between studies and countries with higher and lower income [58]. Generally, women with perinatal depression had total scores of 12 as established by most of the past studies, including the validated Italian EPDS [59] that showed good sensitivity, high specificity, and high positive predictive values (55.6%, 98.9%, and 90.9%, respectively). Therefore, for this study we adopted 12 as cut-off for perinatal depression: <12 Not Perinatal Depression; ≥12 Perinatal Depression.

#### 2.2.2. Offspring

##### Development Quotient, Cognitive and Adaptive Functioning Measures

Children aged under 6 years underwent an evaluation of Development Quotient (DQ) through the administration of the Griffiths III scale. Whereas, for older participants (≥6 years) an evaluation of the Intellectual Quotient (IQ) was performed using the Wechsler Intelligence Scale for Children—Fourth Edition (WISC-IV). All of these measures used the same standard scores (SS = 100) and standard deviations (SD = 15).

Furthermore, an assessment of adaptive skills through the Adaptive Behavior Assessment System—Second Edition (ABAS-II), was performed for all children included in the study.

Griffiths Scales of Child Development—Third Edition

The Griffith III [60] was employed in order to measure the developmental quotient (DQ) of participants aged < 6 years.

This scale measures the overall child’s development, and comprises several developmental activities, grouped in five domains (foundations of learning, language and communication, eye and hand coordination, personal-social-emotional, gross motor skills).

For each subscale, the Griffith-III provides specific scores (developmental age—in months, scaled score, development quotient, percentile) in addition to a comprehensive measure of child’s development (Total DQ). Developmental quotients have a mean of 100 and a standard deviation of 15.

Wechsler Intelligence Scale for Children—Fourth Edition (WISC IV)

The WISC-IV [61] was employed for participants aged ≥ 6 years, in order to measure their Intellectual Quotient (IQ).

This scale provides five main scores for index abilities (Verbal Comprehension Index, Perceptual Reasoning Index, Working Memory Index, Processing Speed Index) and Full Scale IQ. Standard scores are represented by a mean of 100 and a standard deviation of 15.

Adaptive Behavior Assessment System—Second Edition (ABAS-II)

In order to assess their child’s adaptive functioning, parents were administered the checklist Adaptive Behavior Assessment System—Second Edition (ABAS-II) [62].

The ABAS-II provides a parent-report measure of the main skill areas related to development, behavior, and cognitive abilities.

According to their children’s age, caregivers completed the “0–5 years” form or the “5–21 years” form. Specifically, they had to rate their child’s skills (from 0 = “not able to” to 3 = “able to do it and always performs it when needed”) in relation to 10 domains (i.e., communication, use of environment, preschool competences, domestic behavior, health and safety, play, self-care, self-control, social abilities, and motility) which are organized in 3 main skill areas: (1) conceptual (CAD); (2) practical (PAD); (3) social (SAD). In addition, a General Adaptive Composite (GAC) score is given by the sum of scaled scores from the 10 skill areas. Composite scores have a mean of 100 and a standard deviation of 15.

##### Autism Symptoms Measures

All participants were assessed for the presence of autism symptoms through a measure scored by clinicians: The Autism Diagnostic Observation Schedule—Second Edition (ADOS-2). ASD had to be diagnosed according to the Diagnostic and Statistical Manual of Mental Disorders—Fifth Edition (DSM-5) and the ADOS-2 scores.

Autism Diagnostic Observation Schedule—Second Edition (ADOS–2)

The ADOS-2 [63] is a semi-structured observational assessment—gold standard for the measure of autistic symptoms (socio-communicative difficulties, repetitive and restricted behaviors)—organized in different modules (Toddler; Module 1,2,3,4), addressed to a specific participant’s age and expressive language level (ranging from pre-verbal to fluent speech).

In particular, the Toddler Module is administered to toddlers aged between 12 and 30 months (and able to walk for 5 steps independently); Module 1 is administered to children aged over 31 months with preverbal skills; Module 2 to children with non-fluent verbal skills but including sentences; Module 3 is chosen for children and adolescents (under 16 years) with fluent speech; Module 4 is employed for young adolescents and adults.

All the modules provide the ADOS-2 algorithm which includes the following scores: SA = Social Affect, RRB = Restricted and Repetitive Behavior; Total (sum of AS and RRB).

If the total score on the algorithm reaches or exceeds pre-specified cut-off scores, an individual is classified within the Autism diagnosis (for Module 1,2,3 administered to children aged more than 31 months) or risk (for Toddler Module administered to children aged less than 31 months and able to walk independently).

In order to compare scores across different modules, the ADOS-2 Calibrated Severity Score (CSS) was calculated for each participant. The CSS, ranging from 1 to 10 identifies 4 different categories (none, mild, moderate, high) and provides a measure for the level of autism severity. The Toddler Module (administered to children aged between 12 and 30 months) doesn’t provide a CSS score but it identifies a risk category of ASD.

In the present study, 52 participants were administered ADOS-2 and, according to ADOS-2 criteria of administration, 15 children performed Module Toddler and 37 underwent modules 1 or 2 or 3. Whereas, 7 did not undergo ADOS-2 evaluation because they did comply with ADOS-2 criteria (age and walking abilities). However, ASD core symptoms of these 7 children were clinically evaluated according to DSM 5 criteria.

##### Behavioral Measures

Behavioral difficulties of children have been investigated through the administration of the following parental questionnaires.

Child Behavior Checklist (CBCL)

The questionnaire Achenbach Child Behavior Checklist (CBCL) [64] was administered to parent’s participants in order to evaluate the presence of emotional symptoms and behavioral problems of their children.

The “18 months–5 years” or the “6–18 years” CBCL form was employed according to offspring’s age. Caregivers were asked to rate their child’s adverse behavior on a 3 point Likert Scale (0 = not true, 1 = sometimes true, 2 = often true) depending on the frequency of the behavior, with higher scores showing more problematic behavior.

According to the T-scores the behavior is considered as typical (*T* < 65), borderline (*T* = 65–69), and clinically significant (*T* ≥ 70).

The “18 months–5 years” form consists of 110 items organized in 7 Syndrome Scales (Emotionally Reactive, Anxious/Depressed, Somatic Complaints, Withdrawn, Sleep Problems, Attention Problems, Aggressive Behavior). Each scale is organized in 2 main domains: Internalizing and Externalizing Symptoms. Moreover, a Total Behavior score can be calculated.

The “6–18 years” form consists of 113 items grouped in 8 Syndrome Scales (Anxious/Depressed, Withdrawn/Depressed, Somatic Complaints, Social Problems, Thought Problems, Attention Problems, Rule-Breaking Behavior, and Aggressive Behavior). Additionally, in this case, two main domains, Internalizing and Externalizing Symptoms, and a Total Score are provided. According to the age of the participants, the CBCL was administered to a total sample of 47 children.

Conners’ Parents Rating Scale-Long Form

The Conners’ Parent Rating Scale—Revised [65] is a parent report scale which assesses children’s and adolescents’ behavioral difficulties (in particular hyperactivity and inattention).

This scale was administered to parents of children from 3 years of age (N = 35).

Parents are asked to rate their child’s behavior on a 4 points Likert Scale (0 = not true at all, 1 = just a little true, 2 = pretty much true, 3 = very much true).

The Long Form consists of 80 items grouped in 8 subscales (Cognitive Problems, Oppositional, Hyperactivity-Impulsivity, Anxious-Shy, Perfectionism, Social Problems, and Psychosomatic). The test provides an ADHD Index score which permits to detect children at risk for Attention Deficit and Hyperactivity Disorder.

According to the T-scores the behavior is considered as typical (*T* < 60), borderline (*T* = 61–69), and clinically significant (*T* ≥ 70).

##### Parental Stress Measures

In addition, and in the same context of the child clinical assessment (performed with the instruments described below), mothers underwent a standardized evaluation of parental stress.

A stress level related to parenting was measured through the Parental Stress Index Short Form (PSI-SF) [66], a self-report questionnaire of 36 items organized into 3 subscales (12 items each) which specifically evaluates several domains of parental stress: Parental Distress (PD), Parent–Child Dysfunctional Interaction (P-CDI), and Difficult Child (DC). PD subscale evaluates parental feeling of competence, relation with partner, emotional reactivity related to parenting.

P-CDI provides a measure of parental satisfaction in reference to the caregivers’ relation with the child.

DC evaluates difficulties in parenting specifically related to characteristics of the child (behavioral and developmental profile).

In addition to these stress domains, the PSI-SF includes a Total subscale—resulting from the sum of subscales scores-, which provides an indication of the overall stress of a person in the role of a parent. Scores equal or above 90 percent amount to a clinically significant stress for all subscales, except for P-CDI where 85 is already considered a significant cut off.

### 2.3. Statistical Analyses

Independent samples Mann–Whitney U-tests were performed to evaluate depressive symptoms (EPDS) between women; differences in Intelligence Quotient (IQ), Developmental Quotient (DQ), adaptive functioning, behavioral profiles, and autistic symptoms between offspring. Independent samples t-tests were performed to evaluate differences in age of both women and children.

The Pearson χ^2^ test was employed to evaluate differences in degree of education, employment, pharmacological treatment between women and ASD diagnosis between offspring of all groups of mothers. An alpha level of 0.05 was used for all statistical analyses. When performing multiple comparisons (up to 16), we adjusted the *p*-value using the Bonferroni correction. To keep the family-wise error rate at <0.05, the alpha level was thus set at 0.003 for each comparison. The results are reported as means ± SDs if not otherwise specified. All analyses were performed using the Statistical Package for Social Sciences (SPSS) software (Version 25, Inc., Chicago, IL, USA).

## 3. Results

### 3.1. Clinical Summary: WOMEN

A total of 59 women (mean age 36 years) and 59 offspring (mean age 3.5 years) were finally included in the study (Figure 1). Amongst the sample of women, 31 were categorized as women with Perinatal Depression (PD) whereas 28 did not receive diagnosis of PD (NPD).

The group of PD and NPD women did not differ in terms of age at evaluation during pregnancy (*t* = 1.1; *p* = 0.306), degree of education (χ^2^ = 0.436; *p* = 0.804) and employment (χ^2^ = 3.427; *p* = 0.064;). PD and NPD women differed significantly in EPDS score (U = 494.0; *p* ≤ 0.001) (Table 1).

Moreover, whether during pregnancy women had been prescribed psychopharmacological treatment or not, PD women were subcategorized in: PD-NPT = women with Perinatal Depression NOT Pharmacologically Treated; PD-PT = women with Perinatal Depression Pharmacologically Treated. It is necessary to clarify that pregnant women were not randomized to pharmacological treatment but were prescribed psychotropic medication as usual, based on their clinical conditions.

Within the group of PD women, 16 (52%) were prescribed psychopharmacological medication during pregnancy (PD-PT) whilst 15 (48%) did not (PD-NPT). Specifically, 14 women were pharmacologically treated with SSRI, 2 with SSRI + antipsychotics + benzodiazepines. Within NPD no one received psychopharmacological treatment (Table 1).

### 3.2. Clinical Summary: OFFSPRING

The final sample of offspring consisted of 59 children (mean age 3.5 years; 39 males 20 females) (Figure 1). In correspondence with the maternal categorization in PD women and NPD women, children were defined as offspring of mothers with perinatal depression (O-PD) and children not exposed to maternal depression during pregnancy (O-NPD). Moreover, children were sub-categorized based on maternal pharmacotherapy (O-PD-PT, O-PD-NPT).

O-PD and O-NPD did not differ significantly in terms of age at neuropsychiatric evaluation (*t* = 1.1; *p* = 0.306) (Table 1).

Within the subgroup of O-PD, 16 were exposed to maternal psychopharmacological medication during pregnancy (O-PDT) whilst 15 were not (O-PDNT).

### 3.3. Results Objective A: O-PD vs. O-NPD

We firstly investigated the differences in the clinical profile (developmental, cognitive, behavioral, autistic) and parental stress between the group of children exposed (O-PD) and those not exposed (O-NPD) to maternal perinatal depression during pregnancy.

#### 3.3.1. Development, Cognitive and Adaptive Functioning Evaluation

Regarding the Developmental Quotient (DQ)—measured for participants up to 6 years of age—and Intellectual Quotient (IQ)—measured for children aged older than 6 years-, no statistically significant difference was found between O-PD and O-NPD (DQ: U = 232.5; *p* = 0.5) (IQ: U = 22.5; *p* = 1.0) (Table 2). No significant difference emerged between the two groups of children concerning the adaptive functioning as reported by parents through the ABAS-II questionnaire (GAC: U = 267.0, *p* = 0.02; CAD: U = 255.5, *p* = 0.01; SAD: U = 301.5, *p* = 0.1; PAD: U = 270.0, *p* = 0.03) (Table 2).

#### 3.3.2. Autism Symptoms Measures

At the ADOS-2 evaluation in accordance with DSM-5 criteria, no significant difference emerged in terms of ASD diagnosis (children aged more than 31 months who performed the ADOS-2 module 1,2,3) and risk (children aged less than 31 months who performed the ADOS-2 Toddler Module), between the offspring (O- PD) of PD and NPD mothers (O-NPD) (χ^2^ = 0.790; *p* = 0.674).

Nevertheless, in this specific sample, within offspring of PD mothers, 23 did not meet criteria for ASD, 6 were diagnosed ASD and 2 were considered at risk of ASD. Therefore, the absolute risk of an ASD diagnosis or risk of ASD in this subgroup was 25.8% (95% CI: 14.8–37.2). Whereas among children born from NPD women, 24 resulted as no ASD, 3 met criteria for ASD and 1 was considered at risk for ASD. The absolute risk of an ASD diagnosis or risk of ASD in this subgroup was 14.3% (95% CI: 5.1–22.9). Consequently, the Relative Risk (RR) of an ASD diagnosis in children whose mothers presented with depression during pregnancy, compared to those whose mothers did not, was 1.81 (95% CI: 0.61–5.37; *p* = 0.143).

Even when considering the score ADOS-2 CSS (U = 208.5; *p* = 0.7)—provided for the ADOS-2 module 1,2,3- no statistically significant difference emerged between groups (Table 2).

#### 3.3.3. Behavioral Problems Measures

No significant behavioral difficulties emerged between the two groups of children, as reported by the parental questionnaires CBCL (Internalizing: U = 320.0; *p* = 0.2; Externalizing: U = 328.0; *p* = 0.2; Total: U = 353.0 *p* = 0.07) and Conners’ Parents (Oppositional: U = 173.0; *p* = 0.3; Cognitive/Disattention: U = 178.5; *p* = 0.2; Hyperactivity-Impulsivity: U = 168.0; *p* = 0.3; Anxious-Shy: U = 149.0; *p* = 0.8; Perfectionism: U = 154.0; *p* = 0.7; Social Problems: U = 206.5 *p* = 0.02; Psychosomatic: U = 175.0 *p* = 0.2; *ADHD Index*: U = 195.5 *p* = 0.06) (Table 2).

#### 3.3.4. Parental Stress

The parental stress assessment—performed during the offspring neuropsychiatric evaluation and not during pregnancy- showed a significant difference between O-PD and O-NPD in one of the PSI-SF domains (Parental Distress U = 593.5 *p* = < 0.001) but not in the Parent–Child Dysfunctional Interaction (U = 556.5 *p* = 0.006), Difficult Child subscale (U = 430.5 *p* = 0.5) and PSI Total Score (U = 553.0 *p* = 0.007) (Table 2). In particular, mothers categorized as affected by perinatal depression during pregnancy were characterized by significantly higher scores on the PSI-SF scale Parental Distress, which means an increased stress in this subgroup of women in comparison to the women not affected by perinatal depression.

### 3.4. Results Objective B: Perinatal Depression Subgroup: O-PD Treat vs. O-PD No Treat

Secondly within the subgroup of PD mothers and O-PD offspring, we investigated the difference in the developmental, cognitive and behavioral profile between offspring exposed to maternal psychopharmacological treatment (O-PD Treat) in comparison to Offspring NOT exposed to drug treatment during pregnancy (O-PD No Treat).

It is necessary to specify that we did not differentiate the women on the basis of the medication prescribed—because of the reduced sample size—but we considered them in a single subgroup (PD-T).

No significant differences emerged between the two subgroups of PD mothers (PD-T and PD-NT) concerning age at evaluation in pregnancy (*t* = 0.9; *p* = 0.37) and EPDS score (U = 44.5; *p* = 0.6).

In all the scales administered (developmental, cognitive, behavior, autism symptoms), and in the parental stress measure and in the Conners’ questionnaire, no significant difference emerged between subgroups of children (O-PDT; O-PDNT). In Table 3 results of comprehensive scores are reported.

Regarding the Developmental Quotient (DQ)—measured for participants up to 6 years of age—and Intellectual Quotient (IQ)—measured for children aged older than 6 years-, no statistically significant difference was found between O-PDT and O-PDNT (DQ: U = 38.0; *p* = 0.8; IQ U = 36.0; *p* = 0.3) (Table 3).

Additionally, in regard to adaptive functioning evaluated through the ABAS-II questionnaire, no significant results emerged (GAC U = 149.5 *p* = 0.2; CAD U = 152.5 *p* = 0.2; SAD U = 142.0 *p* = 0.4; PAD U = 148.5 *p* = 0.2) (Table 3).

Specifically concerning autism diagnosis within O-PDT, 19% received diagnosis of ASD or emerged at risk for the neurodevelopmental disorder (67% No ASD); whereas within O-PDNT, 33% was diagnosed ASD or at risk for (81% No ASD). Moreover, no difference in autism symptoms’ severity was found (CSS U = 41.0; *p* = 0.2).

Concerning the behavioral profile measured by the parental questionnaire CBCL, no significant differences emerged between O-PDT and O-PDNT (Internalizing: U = 41.5; *p* =0.01; Externalizing: U = 56.0; *p* = 0.1; Total: U = 65.0 *p* = 0.2).

Regarding the questionnaire Conners’ Parents, no significant differences emerged between groups of children (*ADHD Index*: U = 30.0; *p* = 0.2).

Finally, also for parental distress no significant difference emerged between the two subgroups of PD mothers in all the PSI-SF subscales (Parental Distress U = 109.0; *p* = 0.9; Parent–Child Dysfunctional Interaction U = 78.5; *p* = 0.1; Difficult Child U = 124.5; *p* = 0.6; PSI Total Score U = 117.5; *p* = 0.8).

## 4. Discussion

In this study we investigated the impact of maternal perinatal depression on offspring’s cognitive and behavioral development with a focus on the possible increased risk of ASD.

First off, we evaluated whether offspring of mothers with perinatal depression differ—in the developmental/cognitive profile, the socio-communicative and behavioral phenotype—from children of mothers not affected by perinatal depression.

Secondly, we provided a preliminary clinical characterization (developmental and behavioral) of offspring exposed to maternal perinatal depression treated and not treated with pharmacological intervention during pregnancy.

### 4.1. OPD versus OPND: A Comparison of Offspring’s Clinical Phenotypes

The main findings of the study reported no significant difference in the offspring’s clinical phenotype (developmental/cognitive, behavioral, autism symptoms) of women with perinatal depression (OPD) in comparison to children not exposed to maternal perinatal depression (ONPD) at a mean age of 3.5 years.

These preliminary results based on a limited sample size show how, in our research, maternal perinatal depression did not weigh on diagnosis or risk of autism in offspring. However, the group of mothers with PD was characterized by a higher number of children diagnosed with ASD (6 vs. 3) or at risk for ASD (2 vs. 1) in comparison to NPD mothers.

Our results are not in line with most of the observational studies which reported a two-fold increased risk for ASD in offspring of women with PD [48,49,50,51].

This possible inconsistency is of course related to the different sample sizes (ours is significantly smaller in comparison to other studies), but it might also be due to the different methods of ascertainment of maternal illness and children development [26]. In most of the studies [48,49,50,51] both maternal and children diagnosis originated from medical records, registries, or were retrospectively collected by phone calls, with a subsequent possible misdiagnosis. Whereas in our study women were directly evaluated by psychiatrists for the actual presence of PD during pregnancy, and the children underwent a comprehensive neuropsychiatric assessment including the gold standard measure for ASD symptoms: the ADOS-2. Therefore, our results concerning autism symptoms are hardly comparable with other studies. Further research on a larger portion of population which may include the administration of the ADOS-2 is necessary to better investigate the risk of autism in children of PD women.

Nonetheless, in comparison to the prevalent ASD rate estimated by the CDC (1 in 54 children; 18.5 per 1000 in 8-year-olds) [67] we observed a higher number of ASD diagnoses in both groups of mothers (PD and NPD). This may be explained by the fact that most of the women who referred to the Psychiatric Unit and accepted to participate to the study had some developmental concerns regarding their children; whereas women who refused to join the research were not worried about their offspring’s development and behavior.

Regarding developmental and cognitive profiles, we found that offspring quotients were in a normal range of value and did not differ between groups of children (at a mean age of 3.5 years). This is in line with Kurstjens and Wolke [68] who found that postnatal depression *per se* had no adverse effects on cognitive development of children during the first 7 years of life. However, it is non concordant with others [69,70] reporting that chronic maternal depression was associated with lower motor and cognitive performances in early childhood specifically assessed with standardized tools such as Mullen Scale for Early Learning and Bayley Scale.

Concerning behavioral profiles within our sample, externalizing and internalizing symptoms didn’t emerge as characteristic of specific groups of children. This is not concordant with most of the studies reporting an increased risk of child behavioral problems, less mature regulatory behaviors and increased behavioral inhibition among offspring of mothers with perinatal depression [40,41,42,43,44,45]. These conflicting results are however likely attributable to differences in sample size, recruitment strategies, and assessment methods concerning both maternal diagnosis of perinatal depression and evaluation of offspring behavior. In most of the studies, children were in fact assessed via less reliable clinical observation paradigms if compared to standardized instruments such as CBCL and Conners’ Parents questionnaires [40,41]. Whereas according to our findings, Walker and colleagues [71] (conducted on 1452 children; employing the CBCL for measuring behavioral features) suggested that PD is not directly associated with behavioral problems at 4–5 years of age.

Finally, an increased stress related to parenting emerged within mothers affected by perinatal depression in comparison to women not affected, at a mean distance of 3.5 years from delivery. Significant stress emerged in the PSI-SF Parental Distress subscale, which provides a measure for sense of competence, conflict with a partner, social support, restriction, and depression due to parenting (*PD Subscale*). Interestingly, the mothers with PD did not report significant stress in the Difficult Child subscale, which is expected to be affected in the presence of child pathology. This is concordant with the fact that, within our sample, children of perinatal depressed mothers were not characterized by a worser profile in terms of development, cognitive and behavioral features.

Thus, we may speculate that the finding of a superior parental stress in PD mothers could be explained by a possible maternal sense of guilt or responsibility for being affected by a mental health disorder during pregnancy, a premise which could have impacted on their children’s development. Women recruited in this study were in fact aware of the aim of the project.

To the best of our knowledge few studies investigated the parental stress of mothers affected by perinatal depression using the PSI scale [41]. By administrating the questionnaire to mothers at 6 months after delivery, the authors found that maternal depressive and anxiety symptoms (evaluated at the same time of PSI assessment) were related to fewer optimal measures of parenting, in terms of higher stress, lower sense of competence, and lesser social support. However, Feldman’s results, even if in line with ours, are not directly comparable because maternal diagnosis of PD was assessed in our study only during pregnancy.

### 4.2. Perinatal Depression Subgroup Observation: Exposure to Pharmacological Treatment in Pregnancy, What Effects on the Offspring’s Developmental Trajectory?

When considering only the subgroup of women with PD and their respective offspring, even then no difference emerged in the developmental/cognitive and behavioral profile of children exposed or not exposed to maternal psychopharmacological treatment during pregnancy.

Remarkable is our preliminary finding that maternal psycho-pharmacotherapy during pregnancy was not related to an increased risk of autism in offspring. The lack of a higher risk of autism diagnosis is, in fact, non-concordant with the literature [48,49,50,51]. In this case as well, methodological issues may be responsible for such inconsistency.

Interestingly a recent meta-analysis on the topic [72] underlined the actual difficulty in asserting or refuting the implications of a possible risk for ASD within women affected by PD, and pharmacologically treated during pregnancy. Firstly, the authors highlighted that antidepressant exposure in most of the studies was ascertained from prescription databases (this does not provide any information as to whether the women actually took the treatments prescribed during pregnancy). Moreover, the authors raised attention on the need of considering possible confounding factors (age, duration of pathology, period of depression onset, past maternal illness, substance abuse, other prescribed medications), as missing information in most of the studies. Therefore, the increased risk for ASD reported in most of the studies could be related to other factors including maternal habits or substance use in addition to antidepressants.

Our results suggest—at a preliminary level—that exposure to psychopharmacological treatment during pregnancy does not significantly impair the offspring’s developmental and behavioral profile. However, further longitudinal studies on wider samples of women and their offspring—respectively evaluated during pregnancy and at the same stages of development—are necessary to clarify the possible impact of psychopharmacological drugs on child developmental trajectories, and to disentangle the role of depression *per se* from the role of medication.

### 4.3. Strengths and Limits of the Study

The main strength of this study is its longitudinal design. Mothers were clinically evaluated during pregnancy by adult psychiatrists and data were not retrospectively collected employing a database. Children were assessed through an in-depth clinical and comprehensive neuropsychiatric evaluation including standardized instruments for measurements of development, cognitive and behavioral profile, and autism symptoms.

However, there are also several limitations to be considered. The first and possibly most important limitation is related to the fact that mothers who agreed to participate in this study had probably some developmental concerns regarding their offspring (in comparison to women who did not accept to participate). The likelihood of these limitation is highlighted by the high Absolute Risks of ASD presented in the results. Specifically, mothers (belonging to both subgroups, PD and NPD) already suspecting a developmental problem in their offspring, were possibly more likely to agree to undergo an assessment. At present we could not determine the characteristics of those who did not accept to participate in the study. Therefore, a nonresponsive bias analysis could not be performed. Secondly, the sample of both women and children represents a convenience sample; in particular, women were recruited from a clinical service offering psychiatric assessment and help during pregnancy with an increased possibility of finding individuals with PD; women with PD receiving pharmacological treatment during pregnancy, due to the limited sample size, have not been sub-divided based upon the characteristics of the medication (class, dosage and treatment duration).

The sample size is small (59 children). Consequently, beyond the limited power of the study (1- ß = 0.4), it was not yet possible to include in the analyses the many covariates which are likely to influence the complex bio-social interactions determining the outcome in terms of ASD diagnosis. As soon as the sample size will allow, multi-variable analyses will be necessary to appreciate and disentangle these interactions.

Children were assessed at different ages (mean 3.5 years; range 11 months–9 years) and were not evaluated from the first months of life. Therefore, given the characteristics of the study, the observed number of children with ASD cannot be used to estimate ASD prevalence in the population.

## 5. Conclusions

The early clinical characterization of children born from mothers affected by perinatal depression represents an important health issue, aimed to support and provide interventions for pregnant women diagnosed with PD and for their offspring.

In this study we presented the preliminary results of the mental health safeguard project (SOS MOOD)—addressed to women and offspring—developed to support women during pregnancy and post-partum and to early detect child warning signals of a derailed development.

The SOS MOOD project’s preliminary results on a limited sample size suggest that maternal perinatal depression, whether pharmacologically treated or not, is not significantly associated to an increased risk of autism in offspring and does not significantly impair children’s cognitive and behavioral development. Through this study we provide a preliminary picture of developmental trajectories of offspring exposed to maternal perinatal depression. The long-term aim of SOS MOOD project is to perform a prospective study which might allow to detect the possible interactions between the different environmental factors playing a role in the development of ASD.

The pilot observations presented in this work will necessarily require further investigations on wider and more representative samples, so to disentangle the role and interactions between pharmacological treatment and depression, as well as their possible association with ASD.

## Figures and Tables

**Table 1:** Clinical summary of the women an offspring sample.

WOMEN		
	PD (n = 31)	NPD (n = 28)
Evaluation Age in Pregnancy (Mean Age ± SD)	37 ± 5 yrs	35 ± 5 yrs
Degree of Education	Middle School 10% High School 36.7% College 53.3%	Middle School 10.7% High School 28.6% College 60.7%
Employment	Unemployed 40% Employed 60%	Unemployed 18% Employed 82%
EPDS (Mean± SD)	19± 4.3	4.5± 3.8
Pharmacological Treatment	Yes 52% No 48%	No 100 %
OFFSPRING		
	O-PD (n = 31)	O-NPD (n = 28)
Age of Evaluation (Mean Age in months ± SD)	48 ± 28	38 ± 22
Male, Female	18; 13	21; 7
Exposed to psycopharmacological treatment in pregnancy	16	0

Legend: PD = Women with Perinatal Depression; NPD = Non-Perinatal Depressed Women; O-PD = Offspring of Women with Perinatal Depression; O-NPD = Offspring of Non-Perinatal Depressed Women.

**Table 2:** Main results of the comparison between Offspring of Perinatal Depressed Women (O-PD) and Offspring of Non-Perinatal Depressed Women (O-NPD).

	O-PD	O-NPD	U	*p*
***Developmental, Intellectual Skills***				
(Mean ± SD)				
DQ	105 ± 18	100 ± 2	232.5	0.5
IQ	108 ± 17	110 ± 3	22.5	1.0
***Adaptive Functioning (ABAS-II)***				
(Mean ± SD)				
*GAC*	83 ± 23	97 ± 23	267.0	0.02
*CAD*	88 ± 23	103 ± 20	255.5	0.01
*SAD*	90 ± 21	100 ± 21	301.5	0.1
*PAD*	80 ± 23	95 ± 25	270.0	0.03
***Autism Symptoms Measure***				
(Mean ± SD)				
ADOS-2 CSS	2.73 ± 2.4	2.39 ± 2.2	208.5	0.7
***Behavioral Problems Measures***				
(Mean ± SD)				
CBCL_Internalizing Tot	52 ± 12	48 ± 11	320.0	0.2
CBCL_Externalizing Tot	50 ± 10	45 ± 11	328.0	0.2
CBCL_Tot	44 ± 12	39 ± 8	353.0	0.07
Conners’ Parents ADHD Index	63 ± 18	52 ± 14	195.5	0.06
***Parental Stress Measure (PSI-SF)***				
(Mean ± SD)				
Parental Distress	69 ± 30	36 ± 37	593.5	**<0.001**
Parent–Child Dysfunctional Interaction	60 ± 30	38 ± 25	556.5	0.006
Difficult Child	71 ± 30	58 ± 38	430.5	0.5
Total	72 ± 30	50 ± 35	553.0	0.007

Legend: O-PD = Offspring of Women with Perinatal Depression; O-NPD = Offspring of Non-Perinatal Depressed Women; DQ = Developmental Quotient; IQ = Intellectual Quotient; ABAS-II = Adaptive Behavior Assessment System—Second Edition; GAC = General Adaptive Composite score; CAD = Conceptual Adaptive Domain; SAD = Social Adaptive Domain; PAD = Practical Adaptive Domain; ADOS-2_CSS = Autism Diagnostic Observation Schedule—Second Edition Calibrated Severity Score; CBCL = Child Behavior Checklist; PSI-SF = Parental Stress Index Short Form. Highlighted in bold the significant results.

**Table 3:** Main Results of the comparison within PD subgroups, between offspring exposed to maternal psychopharmacological treatment (O-PDT) and not exposed (O-PDNT).

	O-PDT	O-PDNT	U	*p*
***Developmental, Intellectual Skills***				
(Mean ± SD)				
DQ	105	106	38.0	0.8
IQ	112.86	104.63	36.0	0.3
***Adaptive Functioning (ABAS-II)***				
(Mean ± SD)				
GAC	86 ± 22	80 ± 24	149.5	0.2
CAD	92 ± 23	84 ± 23	152.5	0.2
SAD	92 ± 23	87 ± 20	142.0	0.4
PAD	83 ± 23	77 ± 23	148.5	0.2
***Autism Symptoms Measure***				
(Mean ± SD)				
ADOS-2 CSS	2.17 ± 2.32	3.4 ± 2.5	41.0	0.2
***Behavioral Problems Measures***				
(Mean ± SD)				
CBCL_Internalizing Tot	47 ± 11	57 ± 10	41.5	0.01
CBCL_Externalizing Tot	46 ± 8	53 ± 11	56.0	0.1
CBCL_Tot	41 ± 8	48 ± 14	65.0	0.2
Conners’ Parents ADHD Index	59 ± 18	67 ± 18	30.0	0.2
***Parental Stress Measure (PSI-SF)***				
(Mean ± SD)				
Parental Distress	70 ± 29	69 ± 33	109.0	0.9
Parent–Child Dysfunctional Interaction	53 ± 32	68 ± 26	78.5	0.1
Difficult Child	71 ± 31	70 ± 30	124.5	0.6
Total	74 ± 27	70 ± 34	117.5	0.8

Legend: O-PDT = Offspring of Women with Perinatal Depression Pharmacologically Treated; O-PDNT = Offspring of Non-Perinatal Depressed Women Not Pharmacologically Treated; DQ = Developmental Quotient; IQ = Intellectual Quotient; ABAS-II = Adaptive Behavior Assessment System—Second Edition; GAC = General Adaptive Composite score; CAD = conceptual adaptive domain; SAD = Social Adaptive Domain; PAD = Practical Adaptive Domain; ADOS-2_CSS = Autism Diagnostic Observation Schedule—Second Edition Calibrated Severity Score; CBCL = Child Behavior Checklist; PSI-SF = Parental Stress Index Short Form.

## Data Availability

The data presented in this study are contained within the article.
